# The Book World of Medicine and Science

**Published:** 1904-01-30

**Authors:** 


### The Book World of Medicine and Science.

The Nutrition of the Infant. By Ralph Vincent, M.D. (London: Baillière, Tindall and Cox. 1904. Pages 295. Price 10s. 6d.)

Dr. Vincent's book is a work which takes a wider view of the subject of the nutrition of infants than is usually taken in books which deal with infant feeding. That is to say, the author indicates the fundamental principles which should be observed in order to obtain a maximum efficiency of nutrition in each individual case; in other words, the personal index of each infant is taken into consideration, and rule of thumb methods and universal systems of feeding are strongly condemned. The author insists that the only possible way of obtaining consistently good results is to modify the food on a percentage system and to vary the constituents with the physiological indications. The system adopted by the Walker-Gordon Laboratories is favourably regarded by Dr. Vincent, and this system, no doubt, would be generally accepted were it not for the very practical objection that the cost of employing this variety of milk is beyond the reach of any but the well-to-do classes. As a substitute for this expensive method the author supplies full directions for modifying milk at home on the same principle as that employed by the laboratories. This system would answer exceedingly well were the directions easier to carry out in practice, but in order that this may be done, it is necessary to have at hand a 32-per-cent. cream, prepared whey, and a fat-free milk. These ingredients are not easy to obtain, and the method is so complicated that in spite of its undoubted advantages it is unlikely to come into general use. Neither sterilisation nor Pasteurisation is recommended, a somewhat bold position to take up, considering that the method which the author recommends necessitates considerable manipulation of the milk and considerable delay in the preparation. However, this is quite a debatable question, and no doubt if contamination could be avoided it would be far better to dispense with any form of sterilisation. We regard Dr. Vincent's book as a successful exposition of the possibilities and advantages of percentage feeding, and it is the only English work, with which we are familiar, which attempts a systematic description of a home method of percentage modification of milk which provides for the differential graduation of the lactalbumen and caseinogen. But in spite of the undoubted advantages of modifying the percentages of these constituents separately, we fear, in introducing these additional complications in the modification of milk, the author will defeat the very objects which he has in view. Nevertheless, we congratulate Dr. Vincent on his courage and enterprise in attempting to popularise what is undoubtedly a thoroughly scientific and sound system of infant feeding.

A Treatise on Massage. By Douglas Graham, M.D. Third Edition, Revised, Enlarged, and Illustrated. (Philadelphia and London: J. B. Lippincott Co. 1902. Price 21s. Pages, 452.).

There is nothing new under the sun, and certainly there is very little that is original to be said on the subject of massage since the time of Hippocrates who, some 2,000 years ago, wrote his classical account of its uses and applications. Dr. Douglas Graham has, however, managed to collate in a large volume, most of the useful information which has been written on this subject since the papyrus period. He supplies the history of massage, and the latest refinements of the methods employed, and, further, he has attempted the difficult task of teaching by means of illustration and words how to carry out the various methods in practice. The author is himself a masseur of considerable experience, and he maintains that there is nothing derogatory or undignified in a physician utilising the advantages which he possesses by reason of his scientific and professional education in executing the manual processes for himself. No doubt he is quite right in his contentions, but the pecuniary rewards of the professional masseur are hardly such as to offer an adequate return for the heavy expense of a medical education. Massage is perhaps one of the most useful expedients in medicine, but, as is so often the way with mono-enthusiasts, the author has in our opinion exaggerated the virtues of his therapeutic hobby. The book, however, is so complete, and of such a practical character, that we strongly advise all those who are interested in the subject of massage to read Dr. Graham's manual.

Beautiful Biskra. By C. Howard Tripp. Pp. 93. With illustrations. (London: Bemrose and Sons, Limited.)

The best part of Mr. Tripp's little booklet is the illustrations, which are indeed excellent. The photograph of the solitary lion in the frontispiece and "Arabs praying at Biskra" are striking examples. Of the pamphlet itself there is not much to say; it is of the usual guide-book kind, and as the author visited Algeria, Tunis, Sicily, Italy, and the Riviera in five weeks it is patent that he can only have skimmed the surface of things. He remarks how pleasant and profitable such a holiday would be to the overworked man of business, or the valetudinarian in need of sunshine and health; but money, and a good deal of it, is necessary for such a trip. The journey is very long and therefore expensive, and board and lodging is not cheap.

The Riviera and Italy for a £10 Note. (14 Pages. 11 Illustrations. 1 Map. 30 Fleet Street, E.C.)

We have received a little booklet of 14 pages advocating the Newhaven, Dieppe and Paris route to the Continent. The title is "The Riviera and Italy for a £10 Note." This ticket allows 60 days on the Riviera *via* Marseilles to Genoa. The Newhaven and Dieppe route has not in past years been a favourite one, but the disadvantages which once made it unpopular are now entirely removed, and one may leave London in the morning, pass through Paris without a break, and be at Nice the afternoon of the following day. This little pamphlet is issued in the interests of the Brighton and Western of France Companies' Continental service *via* Newhaven and Dieppe, and it is worth while to write for it to 30 Fleet Street, E.C., if kind fate promises a holiday.

Willing's Press Guide, 1904. (Published by Willing's, Limited, 125 Strand, W.C. Price one shilling.)

The thirty-first annual issue of this useful Press Guide fully bears out the reputation of its predecessors as being the handiest and readiest for reference of the many guides published. It forms a very complete directory to the press of the world, and the information it contains can safely be relied upon as being accurate and up-to-date.

